# The effect of intermittent anode potential regimes on the morphology and extracellular matrix composition of electro-active bacteria

**DOI:** 10.1016/j.bioflm.2021.100064

**Published:** 2021-12-13

**Authors:** João Pereira, Yuniki Mediayati, H. Pieter J. van Veelen, Hardy Temmink, Tom Sleutels, Bert Hamelers, Annemiek ter Heijne

**Affiliations:** a Wetsus, European Centre of Excellence for Sustainable Water Technology, Oostergoweg 9, 8911MA, Leeuwarden, the Netherlands; b Environmental Technology, Wageningen University, Bornse Weilanden 9, P.O. Box 17, 6700, AA, Wageningen, the Netherlands

**Keywords:** Bioanode, Intermittent anode potential, Electron storage, EPS formation

## Abstract

Electro-active bacteria (EAB) can form biofilms on an anode (so-called bioanodes), and use the electrode as electron acceptor for oxidation of organics in wastewater. So far, bioanodes have mainly been investigated under a continuous anode potential, but intermittent anode potential has resulted in higher currents and different biofilm morphologies. However, little is known about how intermittent potential influences the electron balance in the anode compartment. In this study, we investigated electron balances of bioanodes at intermittent anode potential regimes. We used a transparent non-capacitive electrode that also allowed for in-situ quantification of the EAB using optical coherence tomography (OCT). We observed comparable current densities between continuous and intermittent bioanodes, and stored charge was similar for all the applied intermittent times (5 mC). Electron balances were further investigated by quantifying Extracellular Polymeric Substances (EPS), by analyzing the elemental composition of biomass, and by quantifying biofilm and planktonic cells. For all tested conditions, a charge balance of the anode compartment showed that more electrons were diverted to planktonic cells than biofilm. Besides, 27–43% of the total charge was detected as soluble EPS in intermittent bioanodes, whereas only 15% was found as soluble EPS in continuous bioanodes. The amount of proteins in the EPS of biofilms was higher for intermittent operated bioanodes (0.21 mg COD proteins mg COD biofilm^−1^) than for continuous operated bioanodes (0.05 mg COD proteins mg COD biofilm^−1^). OCT revealed patchy morphologies for biofilms under intermittent anode potential. Overall, this study helped understanding that the use of a non-capacitive electrode and intermittent anode potential deviated electrons to other processes other than electric current at the electrode by identifying electron sinks in the anolyte and quantifying the accumulation of electrons in the form of EPS.

## Highlights

• Continuous acetate feeding and intermittent anode potential lead to EPS production in electro-active bacteria.
• A charge balance was made including soluble EPS and planktonic cells.
• Patchy biofilm morphologies and more planktonic cells were observed when intermittent anode potential was applied.
• Biofilms grown under intermittent anode potential had more EPS and more proteins.

## Graphical abstract

**Figure:**
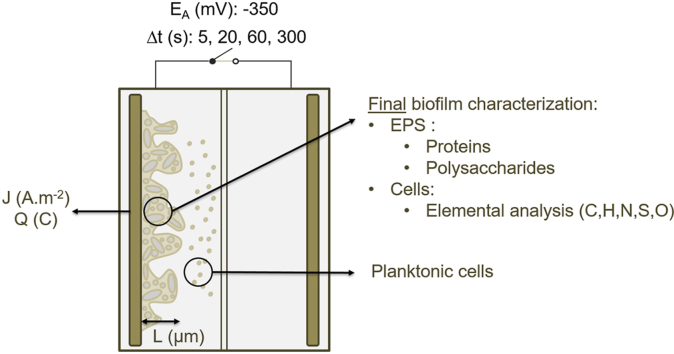

## 1 Introduction

Bio-electrochemical systems (BESs) are environmental biotechnologies with a wide range of applications such as wastewater treatment, nutrient recovery, chemicals synthesis and biosensors [[1], [2], [3]]. The functioning of these systems relies on electro-active bacteria (EAB) that grow on an electrode and planktonic cells. The first performs direct electrons transfer with the anode surface whereas the formed may contribute for electron transfer using soluble redox mediators. These microorganisms in the biofilm catalyze the degradation of organic compounds using the electrode as electron acceptor (at the anode), or they catalyze the synthesis of chemical compounds using the electrode as electron donor (at the cathode).

Bioanodes are the combination of EABs and anodes. Given their ability to combine the oxidation of organic matter with energy recovery, bioanodes form the basis for sustainable wastewater treatment technologies [4]. A key performance parameter of bioanodes is the produced current, which depends on the activity of the electro-active bacteria. The current represents the electron transfer rate to the electrode catalyzed by these EAB and depends, amongst others, on the anode potential [5,6].

A common approach to study EAB is by poising the electrode at a continuous anode potential [7,8]. Instead of continuous anode potential, EABs can also be cultured and studied under intermittent anode potential. Intermittent anode potential regimes consist of alternating the anode potential between open cell potential (OCP) and a controlled potential (also called charge-discharge regime). Higher current densities have been reported when electro-active bacteria are grown under intermittent anode potential regimes [9,10] compared to continuous potential. Until now, studies have focused on the EAB adaptation to this intermittent anode potential regime aiming at linking biofilm composition and morphology to bioanode performance (current, charge recovered and Coulombic efficiency) [11,12]. It has been observed that the biofilm morphology on the electrode changes to a more porous structure when an intermittent potential regime is applied [10,13]. This porous structure has been suggested to result in higher current production in thick biofilms by reducing mass transfer resistances and increasing substrate availability inside the biofilm. The application of intermittent anode potential regimes has also been shown to upregulate the production of extracellular structural components present in the biofilm matrix (for example, *pili* structures and cytochromes) that are important for electron transfer within the biofilm and current production [[14], [15], [16]].

The higher current and the higher recovered charge when bioanodes are operated at intermittent anode potential have also been associated to a continued oxidation of the substrate during charging periods (open cell potential) [17]. During these periods, due to the absence of the electrode as final electron acceptor, it is likely that the electrons generated from substrate oxidation are stored inside the cell [18].

Ter Heijne et al. [18] have suggested two different storage mechanisms for these electrons that are generated at open circuit: (a) electrons are temporarily stored in redox proteins present in the cell membrane (cytochromes and flavins) and released to the electrode after repolarization and (b) electrons are taken up by the cells and accumulated in the form of intracellular storage compounds (for example in the form of polyhydroxyalkanoates) that can later be used as electron donor for bacterial growth [19]. From previous work, it is expected that longer intermittent anode potential times stimulates the formation of intracellular storage compounds [10,19,20]. Some of the compounds formed by bacteria can also be released to the extracellular matrix of the EABs [21,22]. When outside the cell membrane, these compounds are commonly called Extracellular Polymeric Substances (EPS). The synthesis of these EPS also consumes electrons, and their amount and composition typically change as a function of the conditions provided to the biofilms [23]. For example, large EPS production is commonly found in bioprocesses in which bacteria are grown at low nitrogen concentrations but non-limiting carbon concentrations (high C/N ratios) [24].

These storage processes and formation of EPS influences the electron balance in bioanodes. Determining the electron balances at bioanodes is of pivotal importance to tackle electron losses and to divert the majority of the electrons towards the electrode. Thus, electron balances are essential to identify and quantify electron consuming processes other than the desired one. Since the performance of BES relies on the EAB, including their storage processes and planktonic growth in the electron balance is key to understand their adaptation to intermittent anode potential conditions.

In this study, we aim to elucidate the effect of intermittent anode potential on bioanode performance, EAB morphology, and electron balances in the anode compartment. The EAB were grown on a Fluorine Tin Oxide (FTO) electrode and its structure and quantity were monitored in-situ using Optical Coherence Tomography (OCT). Electron balances were made based on the total charge, EPS, amount of biofilm, and number of planktonic cells. Finally, the elemental composition of the biomass was assessed.

## 2 Material and methods

### 2.1 Experimental setup and reactor configuration

The bioelectrochemical reactor used in these experiments has previously been described by Molenaar et al. [25]. The anode compartment was composed of an anode flow channel (33 cm^3^), the anode was a transparent Fluorine-doped tin oxide (FTO) coated glass electrode. The flat and transparent electrode surface with an operating area of 22.3 cm^2^ allowed the real-time monitoring of biofilm growth [25]. Besides, the low capacitance of this electrode allowed the study of electron storage mechanics in the biofilm rather than in the electrode. The current collector (graphite sheet) was placed in contact with the FTO electrode and was surrounding the operating area of the anode flow channel. A bipolar membrane (Ralex PEBPM, MEGA a.s., Czech Republic) was placed between the anode and cathode compartment with the anion side oriented towards the anode and the cation side oriented towards the cathode. A bipolar membrane was used to allow migration of protons to the cathode compartment and hydroxyl groups to the anode compartment. The cathode compartment was composed of a flow channel with the same volume as the anode, and a flat platinum/iridium coated titanium plate (Pt/IrO_2_ 80:20, Magneto special anodes BV, Schiedam, The Netherlands) was used as counter electrode. The projected area of membrane and cathode was 22.3 cm^2^.

Each anode chamber was connected to a feed and recirculation peristaltic pump (Masterflex L/S, Cole-Parmer, Barendrecht, The Netherlands). The feed was pumped at 0.16 mL min^−1^ and both anolyte and catholyte were recirculated at a rate of 60 mL min^−1^. Considering the total volume of anolyte (220 mL), a 23 h hydraulic retention time was obtained. A potentiostat (N-stat d-module, Ivium Technologies, Eindhoven, The Netherlands) was used to poise the anode at −0.35 V vs Ag/AgCl electrode (+0.203 V vs. standard hydrogen electrode; Prosense, Oosterhout, The Netherlands). The reference electrodes were connected to the anode flow channel using Haber–Luggin capillary filled with 3 M KCl solution and positioned in between the FTO electrode and the bipolar membrane. Quick-Coupler valves (Swagelok SS-QC4-D-400, USA) were connected to the tubing of each reactor to avoid oxygen penetration into the system while reactors were disconnected for sampling in the OCT (as described in Molenaar et al. [25]). Reactor temperature was controlled in a climate chamber at 298 K.

### 2.2 Inoculum and electrolyte composition

Reactors were inoculated with a mixture of biomass collected from previous bioanodes operated with acetate as substrate. The anolyte constituted of (g L^−1^): 0.82 NaCH_3_COO, 3.40 KH_2_PO_4_, 4.35 K_2_HPO_4_, 0.1 MgSO_4_.7H_2_O, 0.74 KCl, 0.58 NaCl, 0.28 NH_4_Cl, 0.1 CaCl_2_.2H_2_O, 1 mL of trace metals mixture and 1 mL of vitamins mixture according to DSMZ culture medium 141 [26]. In order to inhibit methanogenesis and only evaluate the effect of intermittent anode potential on the EAB, 1.97 g L^−1^ of BrCH_2_CH_2_SO_3_Na was also added to the medium. The anolyte inflow was continuously sparged with nitrogen before and during the experiments to maintain anaerobic conditions before being pumped to the reactor. The catholyte consisted of 50 mM phosphate buffer solution at pH 7. Nitrogen was continuously sparged into the catholyte recirculation vessel during reactors operation to remove hydrogen.

### 2.3 Experimental design

We tested the effect of intermittent anode potential on electro-active bacteria activity, morphology and composition. The anode potential was controlled at −0.35 V vs Ag/AgCl and open circuits were applied with a duration of 5, 20, 60 and 300 s. The open circuit and close circuit durations were equal, meaning that all intermittently operated reactors were operated with a duty cycle of 0.5. As a control experiment, continuous polarization of the anode at a potential of −0.35 V vs Ag/AgCl was used. An acetate concentration of 0.82 g L^−1^ was used in the influent to feed the microorganisms with non-limiting substrate conditions. All the reactors were operated for approximately 30 days (maximum of 41 days), and each condition was tested in duplicate.

### 2.4 Analysis of the effect of intermittent potential on bioanodes

#### 2.4.1 Acetate consumption

Acetate consumption was determined as the difference between the influent and effluent concentrations. After filtration through 0.45 μm pore-size filter (EMD Millipore SLFH025NS, Barendrecht, The Netherlands), acetate concentration was measured using Ultra-High-Performance Liquid Chromatography (UHPLC) (300 × 7.8 mm Phenomenex Rezex Organic Acid H+ column, Dionex ultimate 3000RS, Thermo Fisher Scientific, The Netherlands) [27]. Samples were taken every 2 or 3 days.

#### 2.4.2 Recovered charge

The recovered charge from each bioanode was calculated by the integration of the current produced over the time the anode was poised at −0.35 V vs Ag/AgCl. For the intermittent experiments, the total recovered charge from the bioanodes was calculated by adding the cumulative charge obtained at the end of a polarization cycle to the first charge data point of the following cycle. Data was recorded every second for the intermittent bioanodes in order to quantify the peak current, and every minute for the continuous bioanodes. The charge stored in the intermittent bioanodes was calculated as a difference between all the charge recovered in a discharging cycle and the charge recovered integrating the last measured current density of that cycle over the whole discharging time.

### 2.5 Bioanode growth and morphology

#### 2.5.1 Visualization of biofilm growth and morphology

Biofilm growth on the bioanode was measured using Optical Coherence Tomography (OCT). The use of this visualization technique as a non-invasive method to quantify the biofilm on an electrode in real time was previously reported by Molenaar et al. [25]. After disconnecting the reactors from the system, both hydraulically and electrically, the OCT was used to scan the FTO electrode at 54 evenly distributed spots. The resulting images were processed using a MATLAB script that isolates and counts the biofilm pixels. The number of pixels was converted to biomass weight (mg COD) using the calibration line reported [25]. Given the use of different operating conditions as the ones used by Molenaar et al. [25]; COD measurements to bioanodes grown at continuous and intermittent anode potential were performed to validate the use of the reported calibration line for biofilm quantification. The data obtained fitted the calibration line. Besides the total amount of biofilm used to monitor the growth, OCT was also used to investigate the effect of intermittent anode potential on the biofilm morphology. Thus, 3D pictures (Field of View (mm) – x=2; y=2; z=0.52 and Pixel size (μm) – x=80.00; y=7.78; z=2.05) of the biofilm structure developed on the electrode were taken throughout the experiment for each condition.

#### 2.5.2 Quantification of cells in the anolyte

The amount of biomass in the anolyle was quantified using flow cytometry. Cells were stained with Sybr Green and Propidium Iodide (SGPI) (Invitrogen, Thermo Scientific, The Netherlands) and the fluorescence intensity was measured with Flow cytometer (EasyCyte HT, Guava, The Netherlands). Phosphate buffer solution was used as negative control.

### 2.6 Final bioanode chemical characterization

#### 2.6.1 Separation of cells and EPS

At the end of the experiment, the biofilm was scraped off the FTO electrode. To separate the biomass from the EPS in the biofilm, an ultrasound-based extraction was used as previously described by Yang et al. [28]. This methodology was reported as the most appropriate to perform a rigorous analysis of the EPS because of its high extraction efficiently and minimal cell lysis. Small modifications to the volumes and centrifugation forces used in the original protocol were included. Briefly, biomass was resuspended in 6 mL of 0.9% NaCl solution and sonicated at 20 kHz for 2 min. Then, samples were horizontally shaken at 150 rpm for 10 min and sonicated again for 2 min. After centrifugation at 10000*g* for 15 min, the supernatant was filtered through a 0.22 μm membrane filter (Nalgene syringe filter, Thermo Scientific, The Netherlands) and labelled as loosely bound EPS (LB-EPS). The pellet was subsequently resuspended in 4 mL of 0.9% NaCl solution, sonicated for 10 min and centrifuged at 10000 g for 20 min. Next, the pellet was washed twice with 1 mL of 0.9% NaCl solution. These supernatants were filtered and labelled as tightly bound EPS (TB-EPS). The anolyte was also harvested at the end of the experiment, centrifuged at 10000 g for 15 min and the obtained supernatant was filtered through a 0.22 μm membrane filter. This fraction was finally labelled as soluble EPS (S-EPS).

#### 2.6.2 Total protein and polysaccharides content present in EPS

The EPS was further investigated for protein and polysaccharide composition. Both proteins and polysaccharides concentration were assessed using colorimetric techniques. Total proteins content was quantified with the Pierce BCA protein Assay Kit (ThermoFisher) using bovine serum albumin (BSA) as standard protein. The polysaccharides content was quantified by means of the phenol-sulphuric acid method and by using glucose as standard sugar [29]. The absorbance of the protein samples was measured at 560 nm whereas the absorbance of the polysaccharides samples was measured at 490 nm (spectrophotometer Victor3 1420 Multilabel Counter, PerkinElmer).

#### 2.6.3 Elemental analysis of dry cells

The biomass component of the biofilm was assessed for the relative percentage of the main biomass building elements: C, H, N, S and O. For an effective quantification of the relative percentage of each element in the microorganisms, the pellet obtained after EPS extraction was washed with mili-Q water to remove the salts from the EPS extraction solution. Cells were afterwards freeze dried and, after this heat treatment, the elemental composition of the cells grown in each bioanode was assessed with the elemental analyzer (Flash Smart, Thermo Scientific, The Netherlands).

#### 2.6.4 Microbial community analysis

The microbial community was analyzed for the reactors operated with an intermittent time of 300 s and continuous anode potential. All experiments were performed in duplicate. Samples to the EABs and planktonic cells were taken at the end of the experiment, as well as the starting culture used to inoculate these reactors. DNA was extracted at the end of the experiment following the method described elsewhere [30]. Briefly, the FastDNA SPIN Kit for Soil (MP Biomedicals, Irvine, CA, USA) was used to disrupt the cell membranes and extract DNA using a binding matrix of silica. Subsequently, DNA purity was verified with Nanodrop (NanoDrop 1000 Spectrophotometer, Thermo Scientific, The Netherlands). Normalized DNA extracts (20 ng mL^−1^) and a negative extraction control were afterwards sent to MrDNA (Shallowater, TX, USA) for library preparation and amplicon sequencing. Briefly, the V4–V5 region of the 16S rRNA gene was amplified in a PCR using primers 515F [31] and 926R [32] and PCR products were pooled in equimolar proportions. Prepared libraries were then sequenced with V3 chemistry to generate 300 bp paired-end reads with an Illumina MiSeq (San Diego, Ca, USA) (detailed information can be found in Appendices).

### 2.7 Calculations

#### 2.7.1 Current density and biomass yield of intermittent and continuous bioanodes

The current produced by the bioanodes over time was recorded for each experiment. Since the shape of the current density curves was not always similar for all experiments, and given the peak currents obtained for the intermittent bioanode, a current density was calculated to allow comparison between the different operating conditions. The current density (A m^−2^) was determined by dividing the total charge recovered by the time the anode was controlled at −0.35 V vs Ag/AgCl (in seconds) and the area of the anode. Normalizing to the closed-circuit time, instead of the time the total time, allows a straightforward comparison between the current of intermittent and continuous anode potential regimes.

Monitoring the amount of biomass over time with OCT also allowed the calculation of biofilm yields. The biofilm yield measures the fraction of electrons derived from acetate oxidation that were used for biomass formation in the bioanode (maintenance and overall anabolic processes). Therefore, this is expressed as the ratio between the amount of biofilm measured on the anode, *biofilm on anode (C)*, and the cumulative acetate converted, *Σ Acetate consumed (C)* (Equation (1)).

(1) Biofilm yield = Biofilm on anode (C) / Σ Acetate consumed (C)

We compared the amount of biofilm on the anode with the current density, since we assume that the contribution of planktonic cells for the overall charge recovery is negligible (no direct electron transfer possible between cells and the anode). We used the biomass yields to study the effect of the anode potential regime as the growth of the biofilm on the electrode is time, current and acetate dependent. Experimentally, we measured different biofilm thicknesses and acetate consumptions for each experiment. However, the measured thickness for biofilms under continuous and intermittent anode potential were in the same range (for example, 16.0 μm for continuous and 16.4 μm for 20 s intermittent) and slightly more acetate was consumed by biofilms under a continuous anode potential.

#### 2.7.2 Charge balance

A charge balance was calculated to identify the effect of intermittent anode potential on the distribution of charge among the electron sinks measured in the reactor. In this simplified charge balance (Equation (2)), we assume that the electrons generated after acetate oxidation can have four different sinks: (i) the anode, measured as charge (Q), (ii) biomass growth on the electrode, X_biofilm_, (iii) biomass growth/biofilm detachment as planktonic cells, X_planktonic_, and (iv) soluble EPS (S, EPS) in the anolyte.

(2) Total charge = Q + X_biofilm_ + X_planktonic_ + S, EPS

Methane formation was not included in the balance given the addition of methanogens inhibitor in the medium, and sulphate reduction was assumed negligible since we used low sulphate concentration (0.1 mM). Oxygen penetration in the system was also monitored with an O_2_ probe (Memosens COS81D, Endress+Hauser, Switzerland). A negligible rate of approximately 1 C.day^−1^ (<1% of the total charge) was lost in oxygen reduction, and no significant O_2_ leakages were registered while sampling the reactors.

In order to get insight on the charge distribution in the anode, each of the electron sinks was represented as a fraction of total charge. These fractions were calculated dividing the cumulative charge measured for each process by the total charge of all four processes. The amount of charge of planktonic cells and soluble EPS were calculated after converting these data to COD. For the planktonic growth, a factor of 2x10^−10^ mg COD planktonic cell^−1^ was used [33] and to convert the mass of polysaccharides and proteins to COD, a calculated value of 1.07 g COD g glucose^−1^ and 1.47 g COD g BSA^−1^ were used, respectively. The COD results were later converted to Coulombs (C) using the molecular weight of O_2_ (32 g mol^−1^), the moles of electrons per mole of O_2_ (4) and Faraday constant (96485 C mol^−1^). The LB and TB EPS fractions were not included in the charge balance since these are quantified in the X_biofilm_ parcel. Detailed information about the calculations performed can be found in appendices.

## 3 Results and discussion

### 3.1 Intermittent anode potential decreased the specific activity and increased the biomass yields compared to continuous anode potential

Table 1 shows the current densities calculated for each condition. The current densities for the continuous bioanodes were similar to the current densities for the intermittent anode potential (1.4 ± 0.1 A m^−2^ for continuous and 1.2 ± 0.1 A m^−2^ for 20 and 60 s). Slightly lower current densities (≈ 0.7 ± 0.1 A m^−2^) were observed for 5 and 300 s intermittent times. At these intermittent times, after reaching a peak, the current dropped faster and stabilized at a lower value compared to 20 and 60 s (examples of current profiles and peak currents can be found in Figs C.1 and C.2). The presented current densities are in the same range as previously reported for studies on bioanodes using FTO as anode [25,34].

**Table 1:** Performance analysis of the bioanodes: current density and biomass yield (results expressed as average ± standard deviation).

Anode potential regime	Current (A m^−2^)	Biomass yield (C_biofilm_ C_acetate_^−1^)
cont	1.4 ± 0.1	0.012 ± 0.000
5 s	0.5 ± 0.2	0.009 ± 0.004
20 s	1.2 ± 0.0	0.020 ± 0.001
60 s	1.5 ± 0.2	0.014 ± 0.001
300 s	0.9 ± 0.0	0.025 ± 0.003

In contrast to what has previously been reported [35], we did not observe more recovered charge for intermittent operation compared to continuous operation. This may be related to the use of FTO electrodes that have a low capacitance and, therefore, do not allow electron storage in the electrode. Instead, electrons can only be stored in the biofilm or used to form EPS. This electron storage in the biofilm can be quantified from the peak currents observed in the current profiles of intermittent bioanodes. These peaks reflect the accumulation of electrons during OCP that are released to the electrode after repolarization (capacitive current). The duration of these peaks was approximately 3 s for all intermittent bioanodes, and the stored charge calculated from these peaks was around 5 mC for all the applied intermittent times. Even though EAB operated in intermittent mode acted as a capacitor and electrons were stored, the small amount of charge stored did not increase the overall recovered charge when compared to the EAB operated in continuous mode, as no clear differences in current density were observed.

Analysis of electron balances in the anodic compartment is crucial to understand how most electrons can be diverted towards electric current. The first electron sink that we analyzed was biofilm growth, and the combination of biofilm quantification from OCT measurements and consumed acetate allowed us to determine biofilm yields. Biofilm yields of the bioanodes at intermittent anode potential were higher compared to the continuous bioanode, except for the 5 s intermittent bioanode (Table 1). An average biomass yield of 0.02 C_biofilm_ C_acetate_^−1^ was found for biofilms grown with intermittent times longer than 20 s, whereas 0.01 C_biofilm_ C_acetate_^−1^ was found for biofilms grown with continuous anode potential. This higher yield for intermittent operation suggests that intermittent anode potential increases anabolic processes of electroactive bacteria for non-capacitive electrodes. The biomass yields are lower, but in the same order of magnitude as the yields of 0.05 and 0.02 g biomass-C per substrate-C reported by Aelterman et al. [20] (at −0.2 V vs Ag/AgCl) and 0.04 g biomass COD per g acetate COD reported by Molenaar et al. [25] (at the same potential used in this study).

### 3.2 More planktonic cells and more soluble EPS in bioanodes operated with intermittent anode potential

Electron balances in the anode compartment allows for analysis of the different electron sinks and designating which of these electron sinks has the largest contribution under the operational regimes. Some studies have focused on and quantified the Coulombic losses due to the presence of alternative electron acceptors on the performance of bioanodes [36,37]. In our experiments, a range in Coulombic efficiency of 30–70% was obtained for all the bioanodes (cumulative charge recovered divided by cumulative charge generated out of acetate consumption), and no clear relation between Coulombic efficiency and operational regimes could be discerned.

**Fig. 1:**
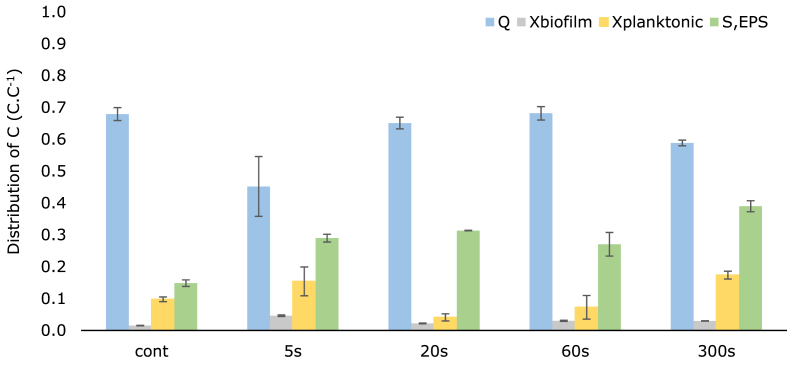
Distribution of charge in the anode compartment: each bar represents the fraction of charge measured in a process normalized by the total charge calculated in the four processes presented.

To compare the electron sinks between operational regimes, we made electron balances by calculating the total charge for the four electron sinks: (i) charge recovered as electric current at the anode, (ii) biofilm, (iii) planktonic cells, and (iv) soluble EPS, and calculated the share of each sink compared to the total charge. Fig. 1 shows the share of each process and how these shares change with the anode potential regime.

Of the four electron sinks, the charge recovered at the anode was the largest fraction for all conditions: higher than 60% for all the bioanodes, except for 5 s intermittent time. This bioanode at 5 s intermittent time showed lower reproducibility due to partial biofilm detachment from the anode registered in one of the replicates, introducing additional variability in current as well as the quantification of planktonic cells.

EPS produced by the bacteria partly remains part of the biofilm and was measured with OCT as biofilm volume (LB and TB EPS are measured as X_biofilm_ in the charge balance). A fraction of these polymers, however, detaches from the biofilm, ending up in solution, referred to as soluble EPS. Interestingly, the second largest electron sink was this soluble EPS: between 27 and 43% of the charge was found as soluble EPS in reactors operated with intermittent anode potential whereas only about 15% of the charge was found as soluble EPS for continuous potential (Fig. 1).

Planktonic cells were the third largest electron sink for all bioanodes, ranging from 5 to 20% of the total charge. Even though there is not a clear trend and effect of intermittent anode potential on the growth of planktonic cells, it is evident that planktonic cells did contribute to the charge balance more than growth of cells in the biofilm for all bioanodes, since the biofilm represented only 2–5% of the total charge. At all operating conditions, the growth of planktonic bacteria that are not involved in direct electron transfer with the electrode appear favored over the growth of bacteria in the biofilm. However, since the presence of alternative electron acceptors in the anolyte is negligible, the presence of planktonic cells is likely a result of detachment of bacteria from the biofilm.

### 3.3 Biofilm and planktonic cells consist of different microbial communities

**Fig. 2:**
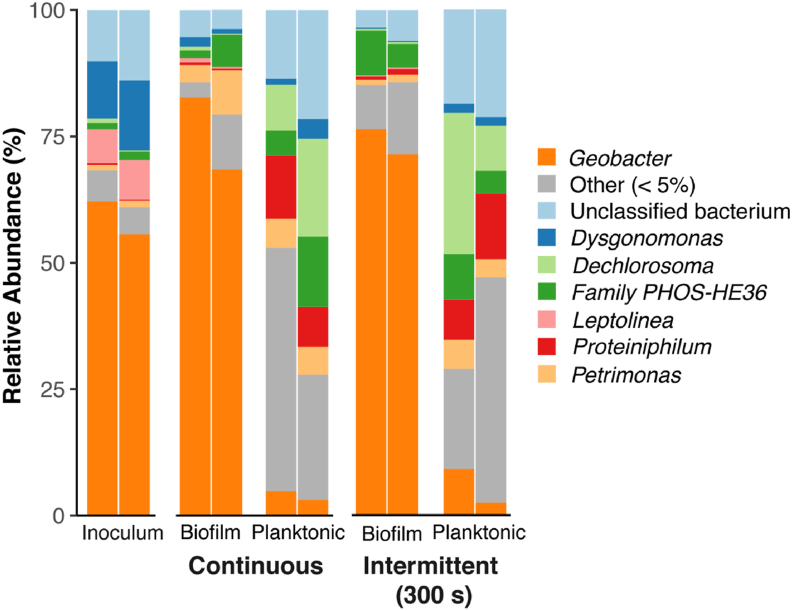
Barplot showing the relative abundances of dominant bacterial genera (>5% on average) in the inoculum, biofilm, and planktonic samples.

Microbial community analysis for the continuous and 300 s reactors revealed a predominance of *Geobacter* in the biofilm communities (approximately 75% of the taxa) and a more diverse microbial composition was found for planktonic bacteria, even though *Geobacter* was still present (less than 10% of *Geobacter*) (Fig. 2; Fig. B.1). Nevertheless, the intermittent (300 s) and continuous anode potential did not affect the microbial community of the biofilm nor the planktonic cells (Fig. 2; Fig. B.1).

The composition of the biofilm communities were similar to the composition of the inoculum. The low abundance of *Geobacter* and the diverse community found as planktonic cells suggest that *Geobacter* grows mainly in direct contact with the electrode surface, and that the top layers of the biofilm, that are more likely to detach, are composed of a more diverse and non-electroactive microbial community. The microbial composition of planktonic cells includes only anaerobic bacteria, indicating that no oxygen leakage occurred in the anode compartment. Among the genera identified, some bacteria may have thriven using alternative electron acceptors other than the electrode. (for example, species from de genus *Dechlorosoma* are known to reduce (per) chlorate [38] whereas the growth of some species of *Petrimonas* is stimulated by the presence of sulphur [39]). Regarding the electron donor, proteolytic bacteria from the genus *Proteiniphilum* are known to use proteins as energy source for growth [40,41]. None of the genera identified contains species well-known for EPS production. More information about the microbial community of the EAB and the planktonic cells can be found in appendices.

### 3.4 Continuous acetate feeding and intermittent control of the anode potential led to more EPS formation

**Fig. 3:**
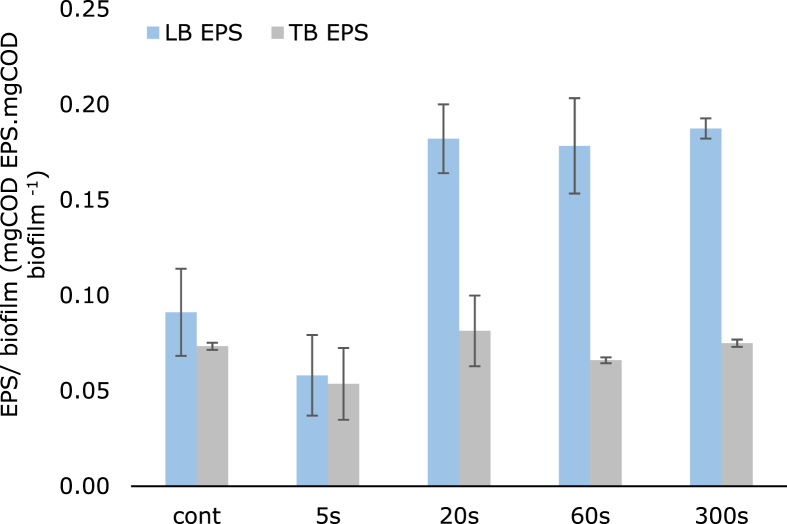
Fraction of LB and TB EPS in the biofilm. The total EPS amount (mg COD) was calculated as a sum of the COD of proteins and polysaccharides measured in the LB and TB EPS. This amount was normalized by the COD of biofilm measured with the OCT. The fraction of EPS is expressed in mg COD protein+polysaccharides mg COD biofilm^−1^.

Anabolic processes include, for example, the synthesis of biomolecules such as proteins and polysaccharides. When under certain conditions, cells tend to produce and release more biomolecules to the extracellular biofilm matrix. The biofilm matrix consists, in addition to microorganisms, of loosely-bound (LB) and tightly-bound (TB) EPS. Here, we quantified the total amount of LB and TB EPS in the electro-active biofilm by adding up the amount of proteins and polysaccharides in each EPS fraction. Since the amount of biofilm on the electrode was different for each experiment, we normalized the EPS produced by the final amount of biofilm measured at the end of each experiment. Fig. 3 demonstrates that the amount of LB-EPS per amount of electro-active biofilms increased when intermittent times longer than 20 s were applied, whereas the EPS fraction tightly bound to the cells was not affected by the polarization regime.

The highest amount of LB EPS of 0.19 mg COD protein+polysaccharides mg COD biofilm^−1^ was found for the biofilm with an intermittent time of 300 s, while the continuous and 5 s intermittent time had the lowest amount of LB EPS of 0.06 mg COD protein+polysaccharides mg COD biofilm^−1^. The results suggest that longer intermittent times favor the use of electrons to synthesize EPS, a metabolic process that is less evident in the biofilms grown under continuous anode potential and in the biofilm grown with an intermittent time of 5 s.

### 3.5 Morphological adaptation of biofilms to anode potential regime

**Fig. 4:**
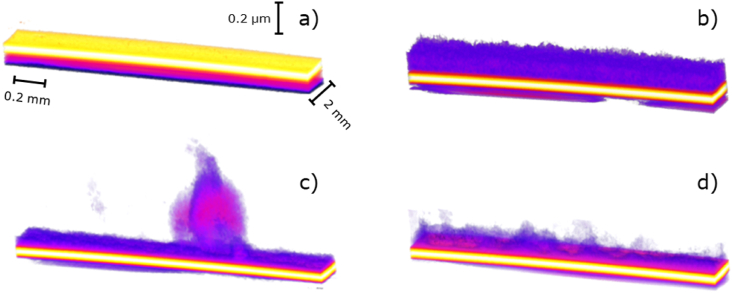
Volume Viewer function in ImageJ was used to visualize the 3D morphology of the biofilm on the electrode: (a) bare electrode, (b) continuous bioanode, (c) 5 s intermittent bioanode and (d) 300 s intermittent bioanode. The yellow structure illustrates the FTO electrode, and the surface where the biofilm grew is depicted on top of this layer. The color underneath the electrode (outside the reactor) is a consequence of partial light reflection on the glass electrode. The scans aim at illustrating exclusively the morphology of the biofilm, and not total amounts. (For interpretation of the references to color in this figure legend, the reader is referred to the Web version of this article.)

OCT was used not only to quantify the biofilm, but also to evaluate the morphological and structural adaptation of the biofilm to the anode potential regime. The OCT images taken of EAB with intermittent anode potential showed irregular shapes whereas more flat and thicker structures were observed in EAB with continuous anode potential. Acknowledging the studies that propose mushroom-like structures as strategies to maximize the biofilm surface area and therefore increase the substrate availably [10,42], these similar structures that we observe in our OCT images in combination with the higher production of LB-EPS suggest that these irregular and loose structures may be clusters of polymers excreted to the extracellular matrix (Fig. 4). The biofilms included in Fig. 4 were scanned at different stages of development.

### 3.6 The amount of proteins in the EPS of biofilms increased in the bioanodes operated intermittently

**Fig. 5:**
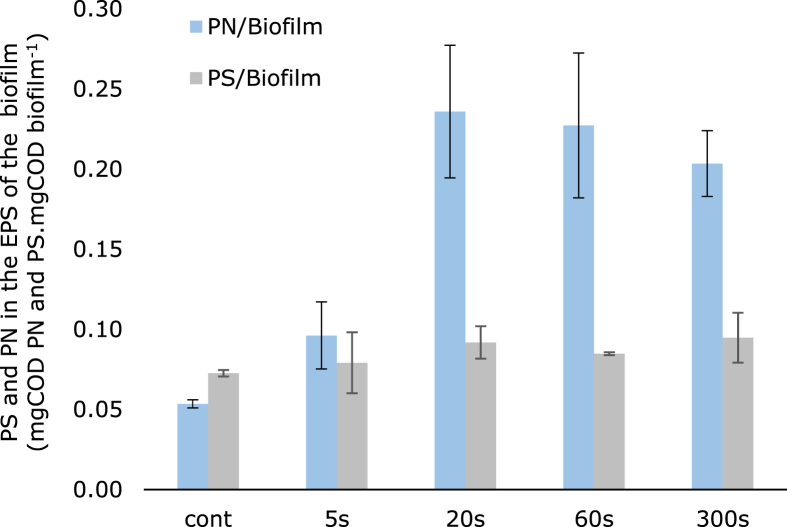
Amount of proteins (PN) and polysaccharides (PS) in the EPS per total amount of biofilm: calculated by dividing the total COD of proteins in the EPS by the total COD of biofilm, and the total COD of polysaccharides in the EPS by the total COD of biofilm (mg COD proteins mg COD biofilm^−1^ and mg COD polysaccharides^−1^ mg COD biofilm^−1^).

To study the effect of intermittent time on the EPS composition of each EAB, the amount of proteins and polysaccharides was measured and normalized to the total amount of biofilm on the anode. Since not all biofilms were grown for the same time nor reached the same thickness, the normalization by the amount of biofilm was used. Fig. 5 shows that more proteins were found in the EPS of EAB that grew under an intermittent anode potential longer than 20 s, while the polysaccharides in the EPS were similar for each condition. The amount of proteins was approximately four times higher when compared with the biofilms under continuous anode potential (≈ 0.21 and 0.05 mg COD proteins mg COD biofilm^−1^, respectively). Overall, the production of proteins was favored over the production of polysaccharides at intermittent times >20 s.

### 3.7 Production of EPS in EABs and the effect of intermittent anode control

EPS production is often related to protection mechanisms in response to unbalanced operating conditions [43,44]. In this study, the stressful condition imposed to the bioanodes was the intermittent availability of the electrode as electron acceptor and the continuous feeding of acetate. The combination of these two parameters challenged the bacteria on finding alternative metabolisms to use the electrons generated after acetate oxidation.

In EABs, a higher amount of proteins is commonly associated to redox-active proteins used in the extracellular electron transfer chain [10]. Yang et al. [28] studied the EPS composition of electro-active biofilms grown at different continuous anode potentials and reported more proteins when biofilms adapt towards the improvement of electron transfer and more polysaccharides as protection mechanism to assure biofilm structure stability. There is thus a balance between protection and electron transfer, reflected in the polysaccharides and protein composition of the EPS.

Since EPS formation facilitates the adhesion of biofilms to surfaces [[45], [46], [47], [48]], the increasing EPS amount with longer intermittent times may have been the strategy adopted by biofilms under intermittent anode potentials to keep their structure attached to the electrode (the electron acceptor). The higher production of EPS in these biofilms to adapt to the anode potential regime corroborates with the higher S EPS concentration in the anolyte of intermittent bioanodes (charge balance in Fig. 1) as the S EPS is a consequence of the release of LB and TB EPS from the biofilm into solution, as well as the washout of cells from the biofilm structure. We neglected the production of EPS from planktonic cells due to the low presence of soluble electrons acceptors and, therefore, lack of energy source. However, the composition of the S EPS is slightly different from the composition of the LB and TB EPS. For example, the ratios PN/PS in continuous bioanode and in the 300 s intermittent bioanode are higher than the ratios PN/PS obtained in the anolyte (≈ 0.4 for the biofilms under continuous anode potential and ≈ 1.6 for the biofilms under intermittent anode potential whereas ≈ 0.02 was found in the S EPS for both experiments). The higher concentration of polysaccharides than proteins in the anolyte indicates that more polysaccharides are released to the anolyte and is also likely to be related to the presence of proteolytic bacteria.

Besides their potential role in extracellular electron transfer, proteins are generally more reduced than polysaccharides. The higher amount of proteins in the EPS of biofilms under intermittent anode potential could be related to the fact that the synthesis of proteins is a more efficient use of electrons in anabolic processes than synthesis of polysaccharides, since more electrons are used to synthesize proteins. Protein synthesis could thus be the strategy adopted by EAB to efficiently use the excess electrons derived from acetate oxidation in periods when the electrode was not available as electron acceptor.

Finally, intermittent bioanodes found the production of EPS an electron consuming mechanism and, at the same time, a strategy to stay attached to the electrode surface.

### 3.8 No effect of the anode potential regime on elemental composition of EAB (dry cells)

Differences in the elemental composition of dry cells could indicate intracellular accumulation of components other than EPS, which may occur as a function of intermittent anode potential control. We used elemental analysis to determine the elemental coefficient of C, H, O, N and S and compared the results to the general biomass formula C_1_H_1.8_O_0.5_N_0.2_S_0.02_ [49]. No difference in elemental composition was observed between the conditions, and coefficients were close to the general biomass formula, which means that the intermittent anode potential does not seem to lead to intracellular accumulation of specific elements (in appendices).

### 3.9 Outlook

EPS production in BES is an unexplored field and its production mechanisms are still to be further understood. Here, we pioneered the study of EPS production in electro-active biofilms as a response to intermittent anode potential regimes by quantifying their production and characterizing their composition in proteins and polysaccharides. The combination of continuous acetate feeding (and no other nutrient limitations) with an intermittent availability of the electron acceptor (non-capacitive FTO electrode), diverted more electrons to EPS production. The production of EPS is an energy consuming process that is often observed in aerobic processes [[50], [51], [52]]. In bioanodes, only the electrode acts as electron acceptor. The only energy yielding processes is acetate oxidation and transfer of electrons through the electron transfer chain towards the electrode. The use of intracellular storage compounds as energy source was not considered, since there was no substrate limitation nor other stress factors (such as nutrient limitation) in the system and therefore no trigger to form intracellular storage compounds. Thus, more studies are needed to decipher the energy source used for the EPS production. Although there are some potential sources of error in the EPS measurements, it is clear that intermittent anode potential led to more EPS production. As an alternative to high ratios C/N required to stimulated EPS production [24], we observed relevant EPS production yields by controlling the anode potential regime intermittently: ≈ 30% EPS in the biofilm and an average of 20% of total EPS recovered from the acetate consumed. In addition to a duty cycle of 0.5, it would be of interest to explore the effects of varying duty cycles on EPS production. Besides, follow-up research is needed for further characterization of the EPS constituents.

Even though the formation of EPS is not the priority application of BES, the results shown here, and the potential practical application of the produced EPS, show the versatility of BES and the value of electro-active bacteria. In the scope of current density enhancement, this study helped understanding that the use of a non-capacitive electrode and intermittent anode potential deviated electrons to other processes other than electric current at the electrode. This knowledge is of key importance to predict electro-active bacteria dynamics in a real wastewater treatment plant where waste streams are continuously fed into the reactor. Testing the influence of intermittent anode potential in the electro-active biofilms morphology and composition using capacitive electrodes is part of the recommended follow-ups for this study. Besides, in a more fundamental study on the effect of intermittent anode potential in EAB, more steps should be taken towards investigating the equilibrium between storage compounds formation and the current production from these compounds. This would unravel the reversibility between formed storage compounds and their use to produce current. More information on these electron consuming processes will allow a more complete and detailed charge balance.

## 4 Conclusion

We studied the effect of intermittent anode potential on the electron balances in the anodic compartment. The composition of the extracellular matrix in which cells are embedded reflected the adaptation of EABs to the applied conditions and provided insights on the fate of electrons in the biofilm. We identified EPS as a major electron sink. In the biofilm, more EPS and more proteins were formed per biomass at intermittent periods >20 s. Besides the biofilm itself, soluble EPS and planktonic growth in the anode compartment represented a considerable fraction of electron sinks. The identification and quantification of these electron sinks is key to tackle losses in the systems and to aim at providing optimum growth operating conditions to increase Coulombic efficiencies of bioanodes.

## Funding

This work was supported by the “Resource Recovery” theme of Wetsus; and 10.13039/501100003246Dutch Research Council (NWO) [project “Understanding and controlling electron flows in electro-active biofilms” with project number 17516 that is part of the research programme Vidi].

## CRediT authorship contribution statement

**João Pereira:** Conceptualization, Methodology, Investigation, Writing – original draft. **Yuniki Mediayati:** Methodology, Validation. **H. Pieter J. van Veelen:** Software, Writing – review & editing. **Hardy Temmink:** Writing – review & editing. **Tom Sleutels:** Conceptualization, Writing – review & editing. **Bert Hamelers:** Conceptualization, Writing – review & editing. **Annemiek ter Heijne:** Conceptualization, Writing – review & editing, Supervision, Project administration, Funding acquisition.

## Declaration of competing interest

The authors declare that they have no known competing financial interests or personal relationships that could have appeared to influence the work reported in this paper.
