# Supplementary material for: The effect of intermittent anode potential regimes on the morphology and extracellular matrix composition of electro-active bacteria
Source: Biofilm. 2021 Dec 13;4:100064. doi: 10.1016/j.bioflm.2021.100064 (PMC8693015; doi:10.1016/j.bioflm.2021.100064)
Supplement: Multimedia component 1 [file mmc1.docx]

**Appendices**

1. **Balance**

The charge balance was calculated using the total recovered charge at the anode, the amount of biofilm measured at the end of the experiment, the total amount of planktonic cells as well as the total soluble EPS. The fraction of charge used for each process was calculated dividing the charge of each process by the total charge measured in four electron consuming processes. Equation A.1 describes the calculation for the fraction of charge recovered at the anode (Q). This was considered the most suitable approach to analyze the data since it directly points out the influence of intermittent potential in the share of each parameter.

Fraction Q = Q / (Q + X_biofilm_ + X_planktonic_ + S, EPS) (Eq. A.1)

For the soluble EPS and the planktonic cells, addition steps were taken to convert these data to Coulombs. For the planktonic cells, a factor of 2x10^-10^ mg COD planktonic cell^-1^ was used to convert the number of cells to COD units (Münch and Pollard 1997), and the COD of glucose (C_6_H_12_O_6_) and BSA (C_123_H_193_N_35_O_37_) were calculated to convert the mass of polysaccharides and proteins to COD units, respectively. The oxidation reactions of glucose and BSA are represented in Equations A.2 and A.3.

C_6_H_12_O_6_ + x O_2_ → 6 CO_2_ + 6 H_2_O (Eq. A.2)

C_123_H_193_N_35_O_37_ + x O_2_ → 123 CO_2_ + ((193-3×35)/2) H_2_O + 35 NH_3_ (Eq. A.3)

We acknowledge that the use of standard composition of sugars and proteins for calculating the charge balance may lead to inaccuracies, since in reality, there may be different sugars and proteins present. Besides, the presence of decay material and other compounds in the EPS may have contributed to interferences in the standard methods used to quantify EPS.

The oxidation coefficient,$x$, represents the moles of O_2_ needed to oxidase the reactant. This parameter was calculated and converted to mass units using the molecular weight of O_2_ (MW_O2_ = 32 g mol^-1^), glucose (MW_glucose_ = 180 g mol^-1^) and BSA (MW_BSA_ = 2751 g mol^-1^). Equation A.4 explains the calculation for the glucose and Equation A.5 for the BSA.

x = (4×6+12-2×6)/4 = 6 mol_COD_ mol_glucose_^-1^ ≅ 1.07 g_COD_ g_glucose_^-1^ (Eq. A.4)

x = (4×123+193-2×37-3×35)/4 = 126 mol_O2_ mol_BSA_^-1^ ≅ 1.47 g_COD_ g_BSA_^-1^  (Eq. A.5)

The COD (mgO_2_ mL^-1^) data were converted to charge (C) using the molecular weight of O_2_ (MW_O2_ =32 g mol^-1^), the moles of electrons per mole of O_2_ (n=4) and Faraday constant (F=96485 C mol^-1^). Since the reactors were operated continuously, the charge used in planktonic cells growth and soluble EPS (Equation A.6) were calculated considering the operating flowrate (Flow=0.16 mL min^-1^) and time between sampling points (∆t = time (d) × 1440).

Charge = COD × (n × F × Flow × ∆t) / MW_O2_ (Eq. A.6)

The total amount of planktonic cells used in the cumulative charge balance was calculated by summing the growth of planktonic cells calculated for every sampling point, whereas the cumulative soluble EPS was calculated assuming a constant concentration of proteins and polysaccharides over the whole experiment time.

1. **Bioinformatics and microbial community analysis (bioanode and planktonic)**

We processed raw 16S rRNA gene sequence data with QIIME2 (v. 2019.10; Bolyen et al. 2019). Briefly, DADA2 (Callahan et al. 2016) was applied to denoise the data and infer exact amplicon sequence variants (ASVs) from error-corrected sequences. We performed quality filtering as follows: primers were removed, sequence length trimmed first at a median quality score of 30 and then for forward and reverse sequences at 240 and 200 bp, respectively. After that, reads were merged, assembled into representative sequences and dereplicated. We then used MAFFT (Katoh and Standley 2013) to align representative sequences, and we applied FastTree2 (Price, Dehal, and Arkin 2010) to construct a bacterial phylogeny. Taxonomic affiliations of representative sequences were predicted using a naïve-Bayesian classification model trained on the SILVA database (v. 132; Quast et al. 2013).

The ASV frequency table, phylogeny and taxonomic information were imported into phyloseq using qiime2R (McMurdie and Holmes 2013; Bisanz 2018) in R (v. 4.0.3; R Core Team 2020). Sequencing of the negative extraction control sample yielded only 479 reads, which was far outside the range of samples (63198 - 152883 reads per sample). No potential contaminant taxa were thus removed from the data prior to downstream analyses. Differences in bacterial community composition (i.e. beta diversity) between inoculum, biofilm and planktonic samples under continuous and intermittent experiments were visualized with principal coordinates analysis (PCoA) from the vegan package (Oksanen et al. 2012). The Bray-Curtis dissimilarity metric was used for this PCoA and was calculated on a feature table containing relative abundances. No rarefaction was applied because all samples leveled off in rarefaction curves (data not shown). Alpha diversity within the communities was assessed using the Shannon diversity index. Bacterial community structure was calculated and visualized at the genus level, highlighting the most dominant genera (average in each set of duplicate samples > 5%). Co-occurrence of genera (> 5% abundance) between biofilm and planktonic samples under continuous and intermittent experiments were visualized using Venn diagrams.


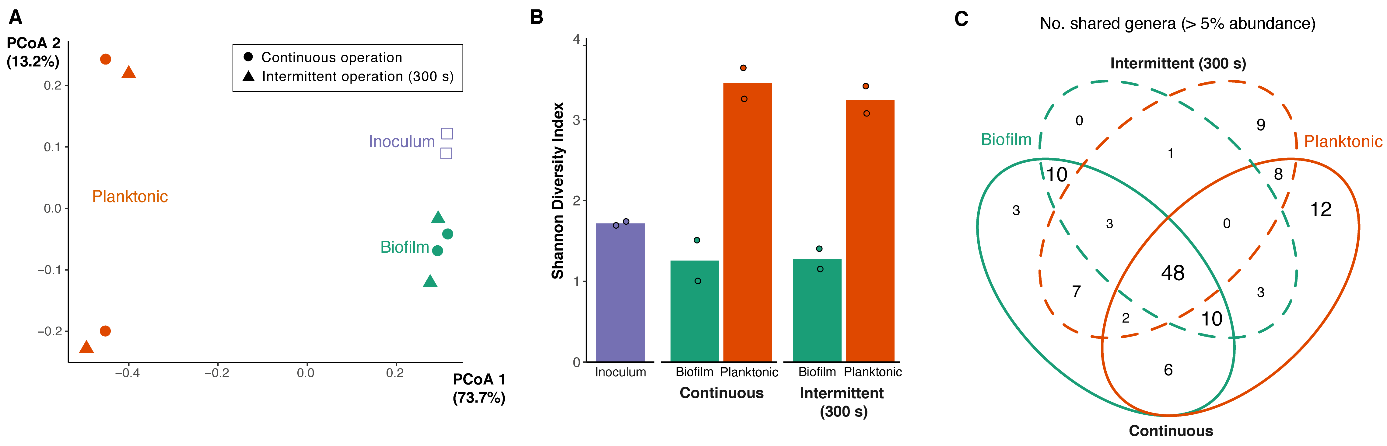


**Figure B.1.** A) Ordination plot showing the variation in bacterial community composition among electrode biofilm and planktonic samples from bioreactors operated in continuous and intermittent modes. Ordination was based on principal coordinates analysis (PCoA) of Bray-Curtis dissimilarities among samples, where each point represents a community and the proximity between points depicts their compositional similarity. B) Barplot showing the median Shannon diversity of two duplicate samples (points) across sample types and operation conditions. Shannon diversity denotes the bacterial diversity within a community and becomes lower when the proportion of rare taxa increases. C) Venn diagram showing the number of co-occurring dominant bacterial genera among sample types and operation conditions. Duplicate runs of the reactors have been inoculated with biofilm (inoculum) formed on an electrode in a previous run. Geobacter was the dominant bacterium in the electrode biofilms. Note that bacterial biomass in planktonic communities is low, and that the presented bacterial community structure represents that low biomass with a minor fraction of Geobacter.

1. **Examples of current densities and demonstrations of peak currents**

Figure C.1 shows examples of the current profiles of both continuous and intermittent bioanodes. The current profiles of the intermittent bioanodes were filtered in order to remove the data points recorded during open circuit (no current). Besides, an average of the currents measured during closed circuit was calculated and used to show the current profile of intermittent experiments over time. An illustration of the peak currents is shown in figure C.2 (experiment at 60 s intermittent time). A peak current followed by a decreasing current (until a stable current was reached) was the current profile observed during closed circuit for all the intermittent times tested.

**Figure C.1.** Examples of the current profiles of the bioanodes operated at continuous and intermittent anode potentials.

Closed-circuit

Open-circuit

**Figure C.2.** Representation of current profiles during closed circuit (discharging time): two consecutive peak currents measured for the bioanode operated at 60 s intermittent time.

1. **Elemental coefficient of the dry cells present in the biofilms of intermittent and continuous bioanodes**

Intracellular accumulation of electrons was studied by performing elemental analysis to the dry cells harvested at the end of each experiment (after EPS extraction). Table D.1 shows the calculated elemental coefficients of the biomass grown under each condition tested.

**Table D.1** Elemental coefficient of the dry cells present in the biofilms of intermittent and continuous bioanodes (results expressed as average ± standard deviation).

|  | C | H | O | N | S |
| --- | --- | --- | --- | --- | --- |
| cont | 1 | 1.69 ± 0.05 | 0.30 ± 0.07 | 0.18 ± 0.01 | 0.01 ± 0.00 |
| 5 s | 1 | 1.59 ± 0.06 | -* | 0.20 ± 0.01 | 0.01 ± 0.00 |
| 20 s | 1 | 1.64 ± 0.02 | 0.31 ± 0.02 | 0.19 ± 0.01 | 0.01 ± 0.00 |
| 60 s | 1 | 1.71 ± 0.01 | 0.31 ± 0.03 | 0.20 ± 0.00 | 0.01 ± 0.00 |
| 300 s | 1 | 1.70 ± 0.04 | 0.33 ± 0.02 | 0.20 ± 0.00 | 0.01 ± 0.00 |

^*^Not enough biomass to analyze the O_2_ content of the cells grown with an intermittent time of 5 s.

1. **References**

Bisanz, Jordan E. 2018. “Qiime2R: Importing QIIME2 Artifacts and Associated Data into R Sessions.”

Bolyen, Evan, Jai Ram Rideout, Matthew R. Dillon, Nicholas A. Bokulich, Christian C. Abnet, Gabriel A. Al-Ghalith, Harriet Alexander, et al. 2019. “Reproducible, Interactive, Scalable and Extensible Microbiome Data Science Using QIIME 2.” *Nature Biotechnology* 37 (8): 852–857. doi:10.1038/s41587-019-0209-9.

Callahan, Benjamin J, Paul J Mcmurdie, Michael J Rosen, Andrew W Han, Amy Jo A Johnson, and Susan P Holmes. 2016. “DADA2 : High-Resolution Sample Inference from Illumina Amplicon Data.” *Nature Methods* 13 (7): 581–583. doi:10.1038/nmeth.3869.

Katoh, Kazutaka, and Daron M. Standley. 2013. “MAFFT Multiple Sequence Alignment Software Version 7: Improvements in Performance and Usability.” *Molecular Biology and Evolution* 30 (4): 772–780. doi:10.1093/molbev/mst010.

McMurdie, Paul J., and Susan Holmes. 2013. “Phyloseq: An R Package for Reproducible Interactive Analysis and Graphics of Microbiome Census Data.” *PLoS ONE* 8 (4): e61217. doi:10.1371/journal.pone.0061217.

Münch, Elisabeth V., and Peter C. Pollard. 1997. “Measuring Bacterial Biomass-COD in Wastewater Containing Particulate Matter.” *Water Research* 31 (10): 2550–2556. doi:10.1016/S0043-1354(97)00089-4.

Oksanen, Author Jari, F Guillaume Blanchet, Roeland Kindt, Pierre Legen-, Peter R Minchin, R B O Hara, Gavin L Simpson, Peter Solymos, and M Henry H Stevens. 2012. “Community Ecology Package.” *… Ecology Package …*, 263. http://mirror.bjtu.edu.cn/cran/web/packages/vegan/vegan.pdf.

Price, Morgan N., Paramvir S. Dehal, and Adam P. Arkin. 2010. “FastTree 2 - Approximately Maximum-Likelihood Trees for Large Alignments.” *PLoS ONE* 5 (3). doi:10.1371/journal.pone.0009490.

Quast, Christian, Elmar Pruesse, Pelin Yilmaz, Jan Gerken, Timmy Schweer, Pablo Yarza, Jörg Peplies, and Frank Oliver Glöckner. 2013. “The SILVA Ribosomal RNA Gene Database Project: Improved Data Processing and Web-Based Tools.” *Nucleic Acids Research* 41 (D1): 590–596. doi:10.1093/nar/gks1219.

R Core Team. 2020. “R: A Language and Environment for Statistical Computing.” Vienna, Austria: R Foundation for Statistical Computing.
